# Batch and continuous fixed bed adsorption of heavy metals removal using activated charcoal from neem (*Azadirachta indica*) leaf powder

**DOI:** 10.1038/s41598-020-72583-6

**Published:** 2020-10-09

**Authors:** Himanshu Patel

**Affiliations:** Department of Applied Science and Humanities, Pacific School of Engineering, Kadodara Palsana Road (NH-8), V: Sanki, Ta. Palsana, Surat, 394305 Gujarat India

**Keywords:** Pollution remediation, Environmental chemistry

## Abstract

The present investigate was intended for adsorption of heavy metals i.e. Pb, Cu, Cr, Zn, Ni and Cd onto activated charcoal prepared from neem leaf powder (AC-NLP) using batch and column studies. Batch adsorption was performed using different variables like adsorbent dose, temperature and contact duration. Thermodynamic analysis of batch treatment concluded that adsorption is thermodynamically feasible and endothermic. This adsorption followed the Pseudo second-order kinetic model derived from correlation coefficient values of chemical kinetic studies. For column study, interpretation of breakthrough curves and parameters were conducted by varying flow rate, initial concentration and bed height; and reveal that optimum conditions were lower flow rate (5 mL/min) and lower initial concentration (5 mg/L) and higher bed height (20 cm). Comparisons of batch and column study through isotherm models were evaluated and column study is more preferred than batch treatment. Maximum Thomas adsorption capacity was achieved upto 205.6, 185.8, 154.5, 133.3, 120.6, 110.9 mg/g for Pb, Cu, Cd, Zn, Ni and Cr respectively. This removal pattern is elucidated by metal ionic properties. Various adsorbing agents such as acids and bases were utilized for adsorption–desorption of AC-NLP.

## Introduction

Water pollution is one of serious problem facing all over the World today, in which wastewater containing heavy metals are continuously released into environment. In few years, there is drastically increasing the ecological and global public health fear connected with atmospheric contamination using these metals. Out of 91 metals, ten metals such as Ni, Co, Mn, Zn, Cr, Cu, Se, Ti and Sb are prime public concern as per World Health organization (WHO)^1^. These metals are available from earth’s crust in very low concentration and found in their metallic, elemental form or chemical bounded with other inorganic materials like carbonate, sulphate, oxide or rock. Metal is considered as a one of the potential pollutant after dye, as it is lethal even at very low concentrations, last long effect, and persistent nature non‐biodegradable i.e. do not undergo microbial or chemical degradation^2^. Due to their highly solubility in aquatic environments, heavy metals are spontaneously immersed by fishes and vegetables. When these metals are come in contact with human, they are penetrating into our body by water, air, food or absorption through skin. Air (industrial and burnt fuel), soil (fertilizer, pesticides, insecticides, etc.) and water (industrial wastewater, rock, etc.) are main sources of heavy metal by which they are accessible in environment^3,4^. These metals are came into environment via various natural processes (volcanic eruptions, saline mist, wild-land fires, disintegration of rock on the Earth surface, biogenic causes and wind-borne soil particles) as well as anthropogenic activities (manufacturing, cultivation, effluent, mining processes, and technological expansion). Out of these, some industries, agriculture and technological expansion are aggressively enhanced the potential exposure onto human significantly. Heavy metal may be affect on nervous system, damage various body organs such as liver, kidney, lungs, brain, and blood, causes Alzheimer disease, etc.^5^. Toxic effects of various metals were reviewed by various scientists^6–8^.

Though several methods such as adsorption, coagulation, membrane, oxidation, biological, chemical precipitation, flotation, ion exchange, and electrochemical deposition with their merits and demerits, but adsorption process is widely utilized for removal of contaminations including metals from raw water^9^. Activated charcoal is used as an adsorbent due to its remarkably high porosity, enhanced pore size and higher adsorption capacities. However due to high cost, it operation is someway inadequate. Currently, investigations are ongoing for preparation of charcoal using low cost materials, natural materials, agricultural waste, etc.^10^. Regarding adsorption process, it is performed by batch, up-flow or down-flow continuous fixed bed, continuous fluidized bed, continuous moving bed and oscillated (pulsed) bed, in which batch and up-flow continuous fixed bed adsorption investigations are more feasible due to easy and cheap technique and can removed most of contaminations^11^. The Neem (scientific name: *Azadirachta indica* and Family Meliaceae—Mahogany) is a large evergreen plant that found most part of Asia and other countries. Its leave, fruits, branches and roots are being used as range of therapeutic treatment such as from controlling digestive disorders to diabetes and high cholesterol to cancer. It is also used as a household pesticide and in toiletries, fertilizer, lubrication, cosmetic, etc.^12^. Previously neem leaf powder was utilized as an adsorbent and corrosion inhibitor^13–16^. The sorption–desorption process is required to reduce solid waste pollution load and cost effective process^9^.

In this paper, the feasibility for adsorption of heavy metals onto AC-NLP was analyzed using batch and fixed bed column study. Several process parameters like temperature, adsorbent dose and contact duration; thermodynamic studies and chemical kinetic analyses were conducted for batch treatment. For column study, breakthrough curves and parameters were accomplished for different initial concentration, flow rate and bed height. Different isotherms were utilized for evaluation the performance by their capacities. Also, experiments of regeneration of AC-NLP were conducted.

## Method and materials

### Material

For batch and column experiments, mature leaves of Neem were collected from near-by area of Pacific School of Engineering, Gujarat. These leaves were thoroughly rinsed with double distilled water to eliminate dust and water soluble impurities, followed by oven dried. The oven dried leaves were powdered and sieved with a < 200 µm to get dried neem leaf powder (NLP). Thereafter, 50 g NLP was impregnated with 200 mL of 1 M phosphoric acid solution with stirred at temperature of 85 °C for 4 h. For preparation of carbonization, impregnated NLP was heated at 330–350 °C in a muffle furnace for 8 h and allowed to cool in desiccators; and used as an adsorbent. The selection of activation agent i.e. phosphoric acid was based on previous experiments conducted by Alau and co-scientists^17^. This AC-NLP was characterized by particle size porosity, pore diameter and pore volume using Mercury Porosimeter and Thermo Quest (Model No.: PASCAL—140). Also, surface area of AC-NLP was analyzed by multipoint Brunauer–Emmett–Teller (BET) model. Degassing was conducted at temperature of 240–250 °C at time duration 10–12 h under vacuum before BET measurement. Experiments were conducted by preparing stock solutions of Cadmium(II), Copper(II), Chromium(II), Zinc(II), Nickel(II) and Lead(II) using CdCl_2_·H_2_O, CuSO_4_·5H_2_O, CrCl_2_·5H_2_O, Zn(NO_3_)_2_·6H_2_O, NiSO_4_·6H_2_O and Pb(NO_3_)_2_ respectively. Thereafter, stock solutions were further diluted as per required of initial concentrations. All the chemicals utilized in these experiments were purchased from Sigma Aldrich and used as it is.

### Experimental details

Batch and column operations were carried out as per Table 1 in triplicates and average values were considered for all these experiments. For batch as well as column study, metal was analyzed before and after experiment as per Standard Methods for Examination of Water and Wastewater^18^. The batch experiments were performed with constant metal concentration (100 mg/L) and stirring speed of 200 rpm. A water bath shaker (Model: 400, Nuve ST), Double beam UV–Vis Spectroscopy (Model: LT-2203, Labman) and Flame Atomic Absorption Spectroscopy (AAS) (Model: PinAAcle 900, Pelkin Elmer) was used for analysis of metals in all the adsorption experiments. In batch experiments, thermodynamic parameters such as change in Gibbs-free energy (ΔG°), enthalpy (ΔH°) and entropy (ΔS°) were determined by differentiating the temperatures (300 to 325 K) and using Langmuir isotherm constant as follows.

$$\Delta {\text{G}}^\circ = - {\text{RT lnK}}_{{\text{L}}} \;{\text{or}}$$

$${\text{InK}}_{{\text{L}}} = - \Delta {\text{G}}^\circ /{\text{RT}} = \, \left( {\Delta {\text{S}}^\circ /{\text{R}}} \right) - \left( {\Delta {\text{H}}^\circ /{\text{RT}}} \right)$$

**Table 1:** Experimental condition for adsorption of heavy metal onto AC-NLP.

Effect of system	Adsorption dose (g/L)	Contact duration (min)	Temperature (K)
**Batch adsorption study**			
Effect of adsorption dose (= W)	2, 4, 6, 8, 10 and 12	120	315
Effect of temperature (= T)	6	120	300, 305, 310, 315, 320 and 325
Effect of contact duration (= t)	6	30, 60, 90, 120, 150 and 180	315

Effect of system	Flow rate (mL/min)	Initial concentration (mg/L)	Bed height (cm)
**Column adsorption study**			
Effect of flow rate (= Q)	5, 10, 15 and 20	50	10
Effect of initial concentration (= C_0_)	10	25, 50, 75 and 100	10
Effect of bed height (= Z)	10	50	5, 10, 15 and 20

**Figure 1:**
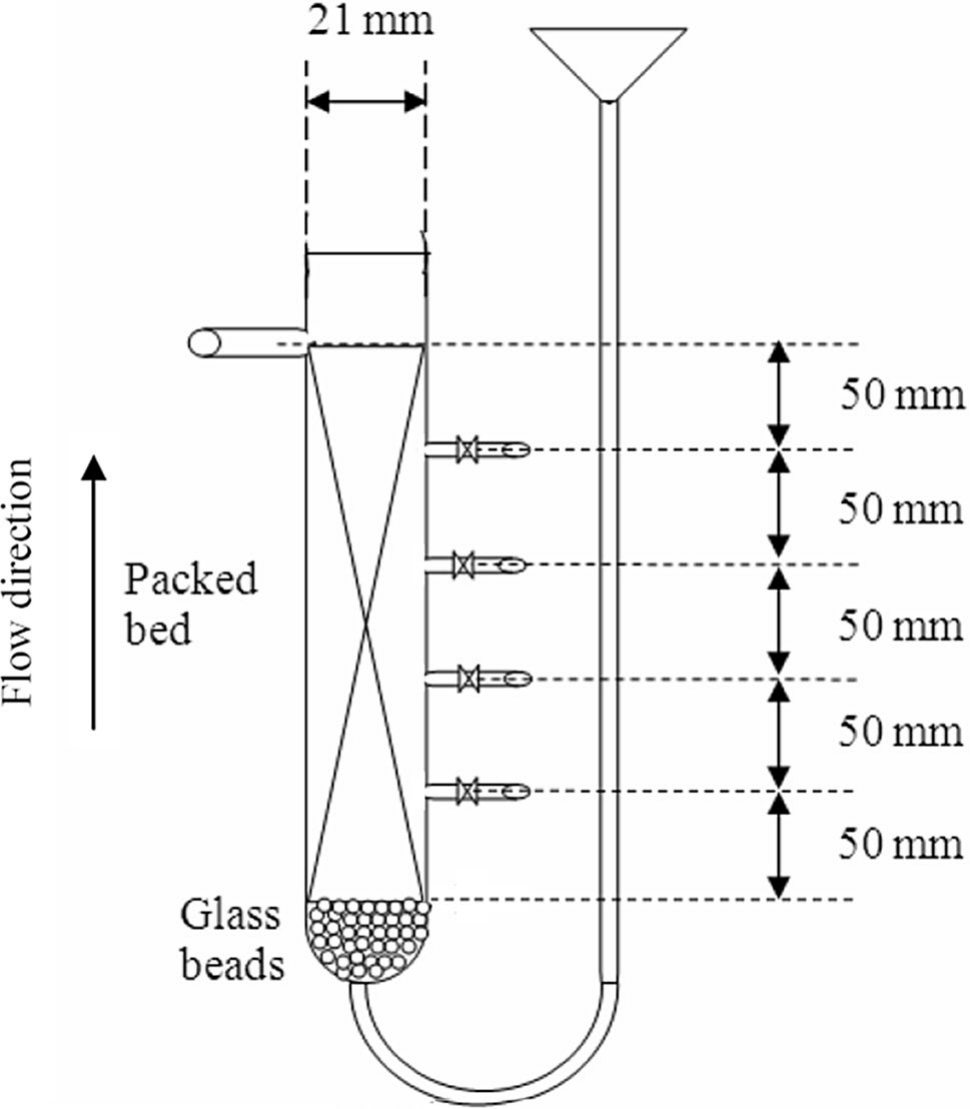
Schematic diagram of fixed bed column used in adsorption study of heavy metals onto AC-NLP.

Straight line graph of ln K_L_ vs. T having slope of ΔH° and the intercept yields the value of ΔS°/T was plotted. The values of these parameters are suggested the important properties such as thermodynamic nature, spontaneity system, type of adsorption (physical or chemical)^19^. Chemical kinetic studies using Pseudo first order, Pseudo second order, Elovich, Intra-particle diffusion model for batch process were also conducted and their correlation coefficients were calculated. The details of kinetic models, importance, their linear equations and plots were previously represented^20^. Further, column study was performed using up-flow continues fixed bed glass column (total length: 30 cm) as per Fig. 1, having with inner radius of 1 cm, packed with 25.5345 g AC-NLP. For consistent flow metal solution flow and avoid channelling, glass bead layer of 2 cm high was prepared at the bottom of column. After glass bead, column equipped with four sampling point at intervals of 5 cm. Metal solution was entered into column from bottom to top mode through peristaltic pump for maintaining desired flow rate.

For column study, various breakthrough parameters are analyzed for establish the efficiency and efficaciously of column study. Table 2 mentioned the breakthrough parameters and its formula for determination^21,22^. To analysis reusability, the sorption–desorption study was carried out onto previously exhausted column of 20 cm with flow rate of 10 mL/min and initial metal concentration of 50 mg/L. Different acids (H_2_SO_4_, HCl, HNO_3_ and H_3_PO_4_) and bases (NaOH, KOH and Na_2_CO_3_) having different concentrations (0.1, 0.5, 1.0, 1.5 and 2 M) were passed though exhausted column at up-flow direction at flow rate of 2 mL/min. The amounts of metal from column outlet were determined. After elution of metal i.e. regeneration of column, it was washed with distilled water at flow rate of 2 mL/min, until pH became 6.5–7.5. Thereafter, adsorption of metal using regenerated column was conducted at flow rate, initial metal concentration and bed height of 10 mL/min, 50 mg/L and 20 cm respectively. This sorption–desorption cycles were conducted for seven times and regeneration efficiency was determined for each cycle using following equation.

$$Regenation\; efficiency \left( \% \right) = \frac{{q_{reg} }}{{q_{org} }} \times 100$$

where, q_reg_ and q_org_ (mg/g) is adsorptive capacity of regenerated column and original capacity of the adsorbent respectively.

**Table 2:** Details of breakthrough parameters.

Sr. no.	Breakthrough parameters	Formula
1	Breakthrough time (t_b_)	Time at C_t_/C_0_ = 0.1
2	Exhaust time (t_e_)	Time at C_t_/C_0_ = 0.9
3	Breakthrough volume (v_b_)	v_b_ = t_b_/Flow rate
4	Exhaust volume (v_e_)	v_e_ = t_e_/Flow rate
5	Time require for the movement of mass transfer zone (t_δ_) downward	$$t_{\delta } = {\raise0.7ex\hbox{${\left( {V_{e} - V_{b} } \right)}$} \!\mathord{\left/ {\vphantom {{\left( {V_{e} - V_{b} } \right)} {Flow\; rate}}}\right.\kern-\nulldelimiterspace} \!\lower0.7ex\hbox{${Flow\; rate}$}}$$
6	Adsorption capacity at t_0.5_ (mg/g)	[Breakthrough time at 50% × flow rate (mL/min) × feed concentration (mg/L)]/mass of adsorbent (gm)
7	Height of MTZ (H_MTZ_)	$$H_{MTZ} = {\raise0.7ex\hbox{${Z \left( {t_{e} - t_{b} } \right)}$} \!\mathord{\left/ {\vphantom {{Z \left( {t_{e} - t_{b} } \right)} {t_{e} }}}\right.\kern-\nulldelimiterspace} \!\lower0.7ex\hbox{${t_{e} }$}}$$
8	MTZ moving rate (U_z_)	$$U_{Z} = {\raise0.7ex\hbox{${H_{MTZ} }$} \!\mathord{\left/ {\vphantom {{H_{MTZ} } {t_{\delta } }}}\right.\kern-\nulldelimiterspace} \!\lower0.7ex\hbox{${t_{\delta } }$}}$$

### Adsorption isotherms

Adsorption isotherm plays vital role as it provide certain useful information such as understanding mechanism of adsorption, surface characteristics and affinity of adsorbate–adsorbent. It also describes the capacity of adsorbent. Various adsorption isotherms are available. The details of most of isotherm models are given previously^23–25^. In batch and column isotherm, graph having straight line was plotted and slop and intercept value of this line was calculated and; thereafter, parameters are calculated.

## Results and discussion

The percentage porosity, particle size, pore diameter, pore volume and surface area of AC-NLP was found to be 41%, 201 mesh, 9.5–9.9 nm, 0.098 cm^3^/g and 612 m^2^/g respectively. Previously, we had utilized NLP as adsorbent and; their particle size and surface area were 122 and 612 m^2^/g respectively. It shows that AC-NLP was increased the surface active sites during charcoal preparation and activation and may be more potential than raw NLP as an adsorbent.

### Batch study

**Figure 2:**
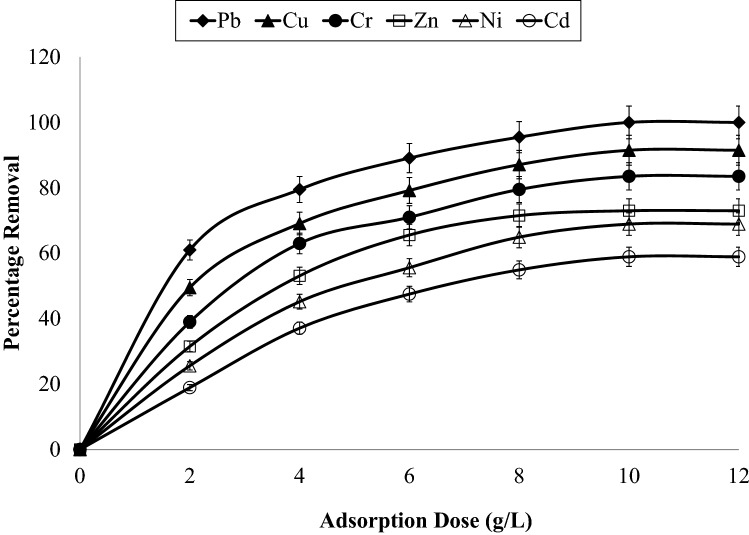
Effect of Adsorption dose on adsorption of heavy metals onto AC-NLP [W: 2, 4, 6, 8, 10 and 12 g/L; t: 120 min; T: 315 K].

Figure 2 represented the effect of adsorbent dose from 2.0 to 12.0 g/L with constant parameters for contact duration (120 min) and temperature (315 K) for adsorption of various heavy metals onto AC-NLP. It shows that percentage removals were increased with increasing dosage of adsorbent upto 10 g/L and then, removals were constant thereafter, which revealed that equilibrium attainted at 10 g/L for heavy metals. This behaviour is because of enhancement in accessibility of surface active sites with increasing dose up to 10 g/L and then, all active sites were exhausted after 10 g/L^26^. Maximum percentage removal of Pb, Cu, Cr, Zn, Ni and Cd was found to be 100, 91.5, 83.5, 73.0, 64.4 and 50.9 respectively using this variable of adsorbent dose. So, order of adsorption capacity for metals are Pb > Cu > Cr > Zn > Ni > Cd. Experiments for evaluate the influence of temperature (300 to 325 K) were conducted at constant adsorbent dose and contact duration of 6 g/L and 120 min respectively for adsorption of heavy metals onto AC-NLP. Percentage removal and other thermodynamic parameters are presented in Table 3 for Lead and Cadmium. Results of percentage removals of other metals are in the midst of Lead and Cadmium. Percentage removal was considerable increased with escalating temperature and highest removal was found at 320 K; and then after equilibrium was attained. Also, negative ΔG° values for all metals indicated thermodynamically feasible and; also, positive value of ΔH° suggested that the adsorption was endothermic and the existence of an energy obstruction like heat in the adsorption process. The positive values of ΔS° show the enhancement of arbitrariness at the adsorbent-adsorbate interface during metal adsorption, which indicated favourable for adsorption.

**Table 3:** Effect of temperature and thermodynamic parameters on adsorption of heavy metals onto AC-NLP [W: 6 g/L; t: 60 min; T: 300, 305, 310, 315, 320 and 325 K].

Heavy metals	Temperature (K)	Percentage removal	Thermodynamic parameters		
			ΔG° (kj/mol)	ΔS° (j/mol K)	ΔH° (kj/mol)
Pb	300	11.2	− 2.22	5.45	245.21
	305	39.6	− 3.05		
	310	59.7	− 3.57		
	315	75.5	− 3.98		
	320	82.4	− 4.59		
	325	82.4	− 5.42		
Cr	300	0.0	− 3.48	8.787	130.45
	305	2.4	− 4.57		
	310	7.0	− 5.45		
	315	27.0	− 7.25		
	320	46.8	− 9.45		
	325	46.8	− 10.25		

Influence of temperature can be explained using following four assumptions: (1) Increasing the temperature may be increase the diffusion rate of adsorbate molecules covering the external boundary layer and in the interior pores of the adsorbent particle, thus decrease in the viscosity of the solution. And therefore, it will alter the equilibrium capacity of the adsorbent and change the equilibrium towards the endothermic path, (2) The mobility of chemicals contributing pollutions like metals may be increasing with increasing the temperature and thus adsorption process is increased, (3) Increasing temperature may be boosts the energy of adsorbate, which lead to more interaction with active sites at the surface, and (4) At higher temperature, swelling effect of inner composition of adsorbent may be occur, which may be increase the penetration ability of adsorbate onto adsorbent^27,28^.

**Figure 3:**
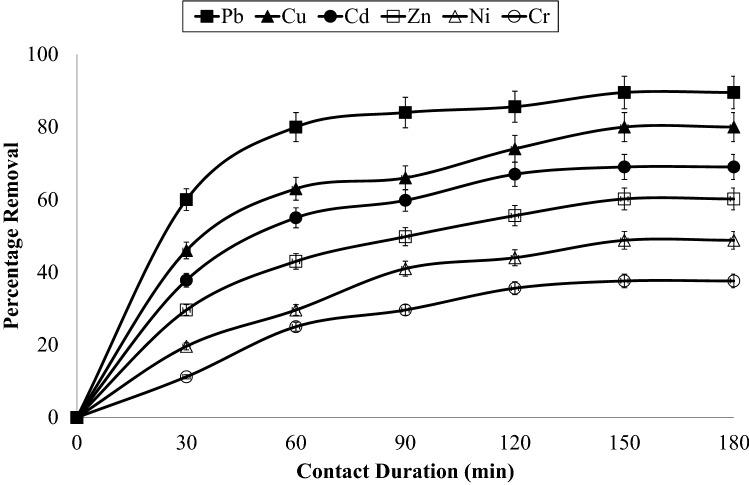
Effect of Contact duration on adsorption of heavy metals onto AC-NLP [W: 6 g/L; t: 30, 60, 90, 120, 150 and 180 min; T: 315 K].

**Figure 4:**
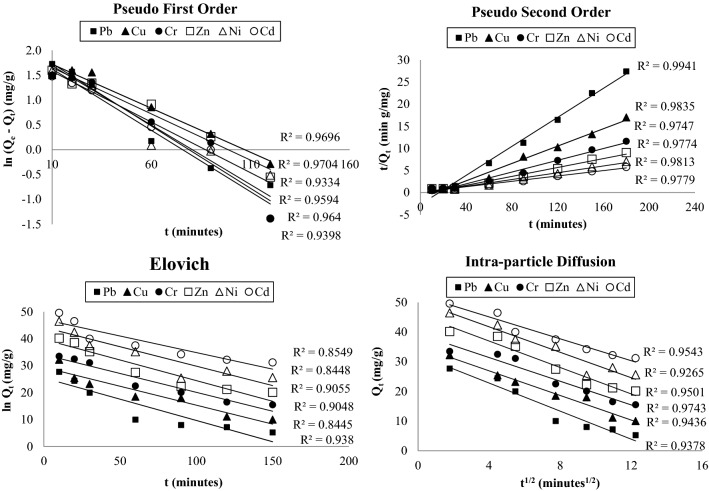
Chemical kinetics curves of heavy metals adsorption by AC-NLP.

The effect of contact duration on metal adsorption at different contact duration namely 30, 60, 90, 120, 150 and 180 min was studied at AC-NLP dose of 6 g/L and temperature of 315 K and results are mentioned in Fig. 3. Preliminary, metal adsorption drastically increased with enlarges with time, thereafter progressively attained the equilibrium. This is probably due to the vacant sites of AC-NLP are more at the beginning of the adsorption process for 90 min^29^. Thereafter, straight line indicated that equilibrium after 90 min contact duration. It indicated that all vacant sites of AC-NLP are exhausted. The plots of chemical kinetics i.e. Pseudo first order, Pseudo second order, Elovich, Intra-particle diffusion model were drawn and mentioned in Fig. 4. Also, their correlation coefficient values (R^2^) were calculated. R^2^ values obtained were 0.970–0.933, 0.994–0.974, 0.938–0.844 and 0.974–0.926 for Pseudo first order [ln(Q_e_ – Q_t_) vs t], Pseudo second order (t/Q_e_ vs t), Elovich (Q_t_ vs t), Intra-particle diffusion model (Q_t_ vs t^1/2^) respectively. It shows that the adsorption of heavy metals onto AC-NLP approaches the Pseudo second order kinetic models. It was expected that metal adsorption of AC-NLP is chemisorptions and rate-limited step. Also, chemical bond is formed between metal species and AC-NLP and contributes to find site that maximize their coordination number with the surface. This phenomenon was previously study adsorption of Indigo Carmine^30^.

### Column study

**Figure 5:**
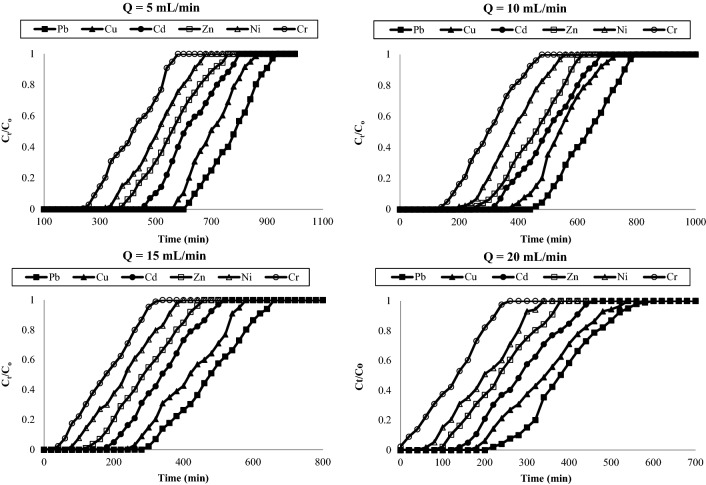
Breakthrough curve for adsorption of heavy metals onto AC-NLP: Effect of flow rate [Q = 5, 10, 15 and 20 mL/min; C_0_ = 50 mg/L, Z = 10 cm].

**Figure 6:**
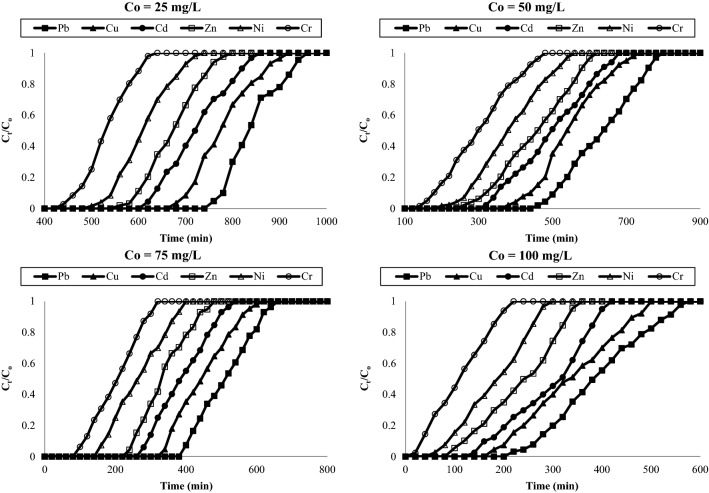
Breakthrough curve for adsorption of heavy metals onto AC-NLP: effect of initial concentration [Q = 10 mL/min; C_0_ = 25, 50, 75 and 100 mg/L, Z = 10 cm].

Figure 5 represented the breakthrough curves for various flow rates i.e. 5, 10, 15 and 20 mL/min for adsorption of heavy metals such as Pb, Cu, Cd, Zn, Ni and Cr onto AC-NLP bed at bed height and initial concentration of 10 cm and 50 mg/L respectively. Effect of initial concentration (25, 50, 75 and 100 mg/L) of various heavy metals with constant flow rate (10 mL/min) and bed height (10 cm) was studies and breakthrough curves were represented in Fig. 6. For both variables i.e. flow rate and initial concentration, breakthrough curves were delayed w.r.t service time and volume. At lower flow rate and initial concentration, adsorption column was difficult to exhaust and; when these parameters were increased, column was easily exhausted. Further, breakthrough parameters for each metal individually were calculated as per Table 2. Table 4 represents these parameters for lead and cadmium, the results of same parameters for other investigated metals i.e. copper, chromium, zinc and nickel were in-between of lead and cadmium. Breakthrough and exhaust time (t_b_ and t_e_); and breakthrough and exhaust volume (v_b_ and v_e_) are decreased with increasing the flow rate and initial concentration. Also, it was observed that height of MTZ was decreasing with increasing flow rate and initial metal concentration. The adsorption capacity at 50% breakthrough capacity was decreased from 62,500.0 to 12,993.42 mg/g with increasing flow rate of metals from 5 to 20 mL/min respectively for Pb. The adsorption capacity at 50% breakthrough capacity was decreased from 21,381.58 to 6907.89 mg/g with increasing flow rate of metals from 5 to 20 mL/min respectively for Cr. Also, The adsorption capacities at 50% breakthrough capacity was decreased from 25,000.0 to 13,651.3 mg/g with increasing initial Pb metals from 25 to 100 mg/L respectively. Also, The adsorption capacities at 50% breakthrough capacity was decreased from 8717.11 to 7236.84 mg/g with increasing initial Cr metals from 25 to 100 mg/L respectively. These statements stated the adsorption capacity was decreasing with increasing flow rate and initial metal concentration.

**Table 4:** Breakthrough parameters for removal of heavy metals using AC-NLP.

Heavy metals	Variables			Breakthrough parameters					
	Q (mL/min)	C_0_ (mg/L)	Z (cm)	t_b_ (min)	t_e_ (min)	t_δ_ (min)	Adsorption capacity at t_0.5_ (mg/g)	H_MTZ_ (cm)	U_z_ (cm/min)
Pb	5	50	10	128	177	9.8	62,500.00	2.77	0.28
	20	50	10	5.6	10.2	0.092	12,993.42	2.51	49.02
	10	25	10	78	93	1.5	25,000.00	2.61	1.08
	10	100	10	28	53	2.5	13,651.32	1.72	1.89
	10	50	5	44	62	1.8	3714.29	1.45	0.81
	10	50	20	65	83	1.8	11,967.21	2.34	2.41
Cr	5	50	10	58	107	9.8	21,381.58	2.58	0.47
	20	50	10	0.5	4.5	0.08	6907.89	2.09	111.11
	10	25	10	47	61	1.4	8717.11	2.42	1.64
	10	100	10	3	19	1.6	7236.84	2.30	5.26
	10	50	5	12	32	2	7540.98	2.13	1.56
	10	50	20	36	58	2.2	15,714.29	2.59	3.45

About flow rate, volume required for exhaustion was extensively increasing with decreasing flow rate. For smaller flow rates, removal efficiency of heavy metal is increases and column was difficult to exhaust. This is due to decrement of contact time between adsorbate and adsorbent, which diminish in adsorption capacity and service time of the bed. Also, effect of flow rate is explanted by mass transfer prerequisites. The rate of mass transfer is increased at higher flow rate and thus, reduces the residence time for metal adsorption onto the surface of AC-NLP. With enhancing the initial concentration of metal, the column is saturate rapidly and thus, decline the breakthrough time^31,32^. The breakthrough: exhaustion time for Pb and Cr achieved at 128:177 and 58:107 min respectively and these times for other metals i.e. Cu, Cd, Zn and Ni were in-between at flow rate, initial metal concentration and bed height of 5 mL/min, 50 mg/L and 10 cm respectively.

**Figure 7:**
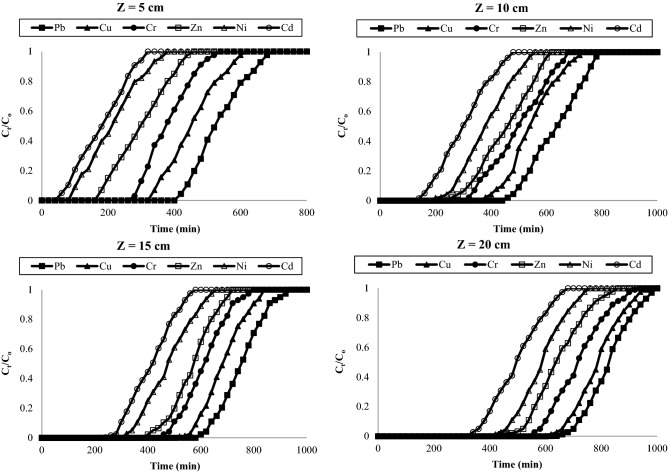
Break through curve for adsorption of heavy metals onto AC-NLP: effect of initial concentration [Q = 10 mL/min; C_0_ = 50 mg/L, Z = 5, 10, 15 and 20 cm].

Breakthrough behaviour of the metal column adsorption process onto AC-NLP at different bed height (5, 10, 15 and 20 cm) with constant inlet metal concentration and of flow rate 10 mg/L and 10 mL/min respectively was investigated and shown as breakthrough curves in Fig. 7. Greater adsorption capacity and hence, higher metal uptake was achieved at higher bed height. Faster breakthrough curves were observed for a bed height of 5 cm, while the slowest breakthrough curve was observed at a bed height of 20 cm. At higher column length, it is difficult to exhaust. Also, breakthrough and exhaust time (t_b_ and t_e_); and breakthrough and exhaust volume (v_b_ and v_e_) are increased with increasing the bed depth. The adsorption capacities at 50% breakthrough are calculated from breakthrough parameters and shown that it was increasing from 3714.29 to 11,967.21 mg/g with increasing bed depth from 5 into 10 cm respectively for lead. And same was increasing from 7540.98 to 15,714.29 mg/g with increasing bed depth from 5 into 10 cm respectively for chromium. This is due increases number of sorption site i.e. total surface area of AC-NLP with in bed height. Moreover, enlargement of mass transfer zone and height of MTZ is due to fact that decreasing slope of breakthrough curve with increasing bed height. The circumstance of axial dispersion in the mass transfer was more potential with decreasing bed height. This reduces the diffusion of the solute which has not enough time to contact and hence, diffuse into entire adsorbent mass^33^. Also, heights of MTZ (H_MTZ_) were below 3 cm, so, fixed bed adsorption of heavy metals onto newly prepared adsorbent is feasible. It is due the fact that column can be functioned over a long time before becoming completely exhausted. From column parameters and breakthrough parameters, it was concluded that lead required the highest time and chromium required least time period to reach the breakthrough concentration that metals, which revealed that adsorption of lead is highest and Cadmium is lowest onto AC-NLP among investigated metals.

### Comparison through isotherm parameters

Table 5 depicted the batch (Langmuir, Freundlich and Temkin) and column (Thomas, BDST and Adam & Bohart) isotherm equations, their respective plots, parameters and their values. Higher correlation coefficient (R^2^) i.e. nearer to one for all models indicates the applicability of all models. For batch isotherm, values of Langmuir affinity constant (K_L_) and Freundlich sorption intensity (n) are between 0 and 1 for all metals, which confirmed the adsorption is favourable. Maximum monolayer uniform adsorption capacity of Langmuir isotherms are 174.7, 154.5, 144.2, 133.4, 122.5 and 111.5 mg/g for Pb, Cu, Cd, Zn, Ni and Cr respectively. Temkin model indicates the nature of adsorption is endothermic and heat required for enhancement of adsorption as Temkin parameter related to the heat adsorption b (J/mol) > O. From correlation coefficient (R^2^) mentioned in Table 3, it is clear that Langmuir model (0.9910 ≤ R^2^ ≤ 0.9948) was closely fits to experimental data than other investigated model i.e. Freundlich model (0.9810 ≤ R^2^ ≤ 0.9884) and Temkin model (0.9721 ≤ R^2^ ≤ 0.9762), which confirmed that Langmuir model was significantly expressed metal adsorption onto AC-NLP. However, Langmuir model is depends on presumption of identical adsorption heat and monolayer adsorption. So, it suggests the monolayer coverage of metals on the flat surface of the AC-NLP having homogeneous distribution of active sites available for sorption on the adsorbent surface. For column study, optimum parameters were selected i.e. lower flow rate (5 mL/min) and initial concentration (5 mg/L) and higher bed height (20 cm) derived from column parameters and breakthrough variables for adsorption of heavy metals i.e. Pb, Cu, Cd, Zn, Ni and Cr onto AC-NLP. So, these parameters were selected for column isotherm study. Maximum Thomas adsorption capacity were achieved up to 205.6, 185.8, 154.5, 133.3, 120.6 and 110.9 mg/g for heavy metals, Pb, Cu, Cd, Zn, Ni and Cr respectively. Derived from correlation coefficient (R^2^), Thomas model is more preferred than other investigated isotherms such as BDST and; Adam & Bohart model, which indicates that follow second-order reversible reaction kinetics and also no axial dispersion. Table 6 lists the comparison the maximum adsorption capacity related Langmuir and Thomas isotherm for removal of metals onto different adsorbents using batch and column study respectively, as per author knowledge. Experiments, mentioned in this table, were conducted for comparison of batch and column study for adsorption of metal, same as per this experiment. Highest adsorption capacities of metal were achieved in this experiment except zinc using batch (470 mg/g) and column study (498 mg/g); and nickel using batch study (147.2 mg/g).

**Table 5:** Batch and column adsorption models and its parameters.

Adsorption model	Linear equation	Plot	Parameters of model	Heavy metals					
				Pb	Cu	Cd	Zn	Ni	Cr
**Batch isotherm**									
Langmuir	$\frac{1}{{q_{e} }} = \frac{1}{{q_{m} K_{L} C_{e} }} + \frac{1}{{q_{m} }}$	$\frac{1}{{q_{e} }} vs. \frac{1}{{C_{e} }}$	K_L_ (L/mg)	0.0678	0.0604	0.0554	0.0454	0.0401	0.0381
			q_m_ (mg/g)	174.7	154.5	144.2	133.4	122.5	111.5
			R^2^	0.9984	0.9985	0.9963	0.9987	0.9948	0.9981
Freundlich	$\log q_{e} = \log K_{F} + \frac{1}{n}\log C_{e}$	$\log q_{e} vs.\log C_{e}$	K_F_ (L/mg)	19.2514	17.4514	15.1424	10.1578	7.1212	6.0287
			n (-)	0.2451	0.3125	0.3525	0.3747	0.4001	0.4212
			R^2^	0.9875	0.9884	0.9825	0.9810	0.9840	0.9864
Temkin isotherm	$q_{e} = \frac{RT}{b}\ln K_{T} - \frac{RT}{b}\ln C_{e}$	$q_{e} vs. \ln C_{e}$	b (kJ/mol)	750.25	725.12	645.45	640.12	632.2	603.1
			K_T_ (L/gm)	45.45	56.45	75.45	80.12	85.12	89.23
			R^2^	0.9758	0.9721	0.9756	0.9752	0.9745	0.9762
**Column isotherm**									
Thomas model	$$\ln \left[ {\left( {\frac{{C_{O} }}{{C_{t} }}} \right) - 1} \right] = \frac{{k_{TH} q_{o} m}}{Q} - \frac{{k_{TH} C_{o } V_{eff} }}{Q}$$	$$ln\left[ {\left( {{\raise0.7ex\hbox{${C_{o} }$} \!\mathord{\left/ {\vphantom {{C_{o} } {C_{t} }}}\right.\kern-\nulldelimiterspace} \!\lower0.7ex\hbox{${C_{t} }$}}} \right) - 1} \right]$$ vs. time	k_TH_ (mL/mg·min)	0.6112	0.5124	0.5714	0.6555	0.5457	0.5252
			q_0_ (mg/g)	205.6	185.8	154.5	133.3	120.6	110.9
			R^2^	0.9858	0.9812	0.9898	0.9878	0.9852	0.9811
Bed depth service time model	$t = \frac{{N_{O} }}{{C_{0} U}}Z - \frac{1}{{k_{BDST} C_{0} }}\ln \left( {\frac{{C_{0} }}{{C_{t} }} - 1} \right)$	Bed-height vs. time	k_BDST_ (mL/mg·min)	0.3787	0.3353	0.3232	0.3425	0.2785	0.3011
			N_o_ (mg/g)	9784.5	9524.5	9455.5	9335.6	9248.0	9025.3
			R^2^	0.9788	0.9699	0.9787	0.9785	0.9758	0.9785
Adam and Bohart model	$$ln\left[ {\left( {\frac{{C_{O} }}{{C_{t} }}} \right) - 1} \right] = K_{AB} N_{O} \frac{Z}{u} - K_{AB} C_{t} t$$	$$ln\left[ {\left( {{\raise0.7ex\hbox{${C_{o} }$} \!\mathord{\left/ {\vphantom {{C_{o} } {C_{t} }}}\right.\kern-\nulldelimiterspace} \!\lower0.7ex\hbox{${C_{t} }$}}} \right) - 1} \right]$$ vs. time	k_AB_ × 10^–5^ (1/mg·min)	8.7822	7.4521	8.0127	7.8784	6.4510	6.2122
			N_o_ (mg/L)	9557.5	9218.2	9025.2	8956.1	8356.9	8124.5
			R^2^	0.9757	0.9787	0.9752	0.9788	0.9745	0.9756

**Table 6:** Comparison of metal removal with previous experiments.

Sr. no.	Adsorbent	Adsorbate	Langmuir isotherm capacity (mg/g)	Thomas isotherm capacity (mg/g)	References
1	Hibiscus Cannabinus kenaf	Cr(VI)	582.3	21.48	^34^
2	Immobilied green microalga, *Synechococcus* sp.	Cd(II)	57.76	74.87	^35^
3	GAC from coconut shell	Pb(II)	29.44	525.0	^36^
4	Tamarind fruit shell	Cu(II)	80.01	110.47	^37^
5	CNT coated PAMAM	Zn(II)	470	498	^18^
6	Electric Arc furnace slag	Ni(II)	147.2	69.9	^38^
7	Activated charcoal from NLP	Pb(II), Cu(II), Cd(II), Zn(II), Ni(II) and Cr(II)	174.7, 154.5, 144.2, 133.4, 122.5 and 111.5	205.6, 185.8, 154.5, 133.3, 120.6 and 110.9	In this study

From batch as well as column study, order of removal for heavy metals was lead > copper > Cadmium > Zinc > Nickel > Chromium. This trend of removals is observed due to mobility of heavy metal and thereafter, their ionic properties in the aqueous phase, because adsorption is also depends upon various ionic properties such electronegativity, ionic radius, hydration radius, degree of hydration, ionic strength, etc. The electronegativies of Pb, Cu, Cd, Zn, Ni and Cr are 1.90, 1.87, 1.65, 1.91, 1.69 and 1.66 respectively. More electronegativity tents higher cation ion exchange at the surface and thus, enhances the adsorption. Also, ion exchange process also depends upon their degree of hydration and hydration radius. The degree of hydration and hydration radius of Pb^+2^ is lower; hence adsorption of lead is highest. Further, order of ionic radius: Pb^+2^ (120 pm) > Cd^+2^ (95 pm) > Zn^+2^ (74 pm) > Cu^+2^ (73 pm) > Ni^+2^ (69 pm) > Cr^+2^ (63 pm). Due to steric overcrowding on the surface, large ionic radius encourages a quick saturation of adsorption sites. As the lead ion (Pb^+2^) has higher ionic radii, so, lead has maximum adsorption capacity compared to other metal ions. While increasing ionic strength, the distribution coefficients (K_D_) are decreases and initial metal concentrations of metals are increases, which trends to reduction in adsorption process. The value of K_D_ for lead is lowest than other investigated metals, so, adsorption of lead is higher.

Among acids and bases having different concentrations, all acids (i.e. low pH) particularly, 0.5 M HCl was found more effective for seven successive sorption–desorption cycles for all instigated metals (Pb, Cu, Cd, Zn, Ni and Cr) using AC-NLP. Suitability of acid was explained by proton exchange principle, which stated that higher number of protons were required for desorption of cationic heavy metals, thus, release the deposited metal from AC-NLP (or replace the bound metal ions from AC-NLP) and; regenerate of AC-NLP. So, regeneration capacity of acid was very high and that of alkali was least. Further, higher concentration of HCl (1.0–2.0 M) and strong acid (H_2_SO_4_) may be destroyed the structure of exhausted AC-NLP, and lower concentration (0.1 M) and weak acid (H_3_PO_4_, HNO_3_) required more time and volume to regenerate the AC-NLP due to may be strong bond formation between heavy metal and AC-NLP. From sorption–desorption data, it was indicated that regeneration efficiencies was slow decreasing from 98.2 ± 3.5 to 65.9 ± 4.9% for all metals in consecutive seven cycles. Also, weight loss of 2% was found in each cycle. It implied that adsorption of heavy metals onto AC-NLP was reversible process, as per discussed in chemical kinetic study. Further, total regeneration was not achieved due to adsorption was chemisorption and strong bond was formed between heavy metal and AC-NLP during adsorption; and not disintegrated even strong acid, hydrochloric acid^39,40^.

## Conclusion

Batch and column adsorption study is conducted for removal of heavy metals such as Pb, Cu, Cd, Zn, Ni and Cr using activated charcoal prepared from neem leaf powder; and following conclusions were made.

1. The batch adsorption was performed using various process variables such as adsorbent dose, temperature and contact duration and found that order of efficiency of these parameters were: adsorbent dose > temperature > contact duration. Maximum removals of metals were found at adsorbent dose of 10 mg/L, contact duration of 120 min and temperature of 315 K.
2. From thermodynamic parameters, it was observed that adsorption is spontaneous (thermodynamically feasible), endothermic and favourable.
3. From correlation coefficient values (R^2^), order of chemical kinetin model for pertinence using adsorption data was Pseudo second order > Intra-particle diffusion model > Pseudo first order > Elovich. It suggested that adsorption follows the Pseudo second order kinetic models, considering that adsorption was chemisorption and rate-limited step.
4. Breakthrough curves for different factors such as flow rate, bed height and initial concentration of metal in column study were plotted and found that adsorption capacity was increasing with decreasing flow rate and initial metal concentration; and increasing bed height. Breakthrough parameters were also calculated which show that fixed bed adsorption of heavy metals onto newly prepared adsorbent was feasible. From breakthrough curves and parameters, optimum parameters were found to be lower flow rate (5 mL/min) and initial concentration (5 mg/L) and higher bed height (20 cm).
5. Parameters including maximum adsorption capacity of batch (Langmuir, Freundlich and Temkin) and column (Thomas, BDST and Adam & Bohart) isotherm were calculated. Langmuir and Thomas isotherm was more convenient model that other investigated models for batch and column model respectively, derived from R^2^ values.
6. The order of metal removals was Pb > Cu > Cd > Zn > Ni > Cr, which was explained using various ionic parameters of metals.
7. Regeneration efficiencies of AC-NLP was achieved from 98.2 ± 3.5 to 65.9 ± 4.9% in consecutive seven cycles using 0.1 M HCl, which concluded that adsorption was reversible and chemisorption.
